# Analysis of deafness susceptibility gene of neonates in northern Guangdong, China

**DOI:** 10.1038/s41598-023-49530-2

**Published:** 2024-01-03

**Authors:** Zhanzhong Ma, Wenbo Huang, Jing Xu, Jianwu Qiu, Yulan Liu, Meixian Ye, Shushu Fan

**Affiliations:** 1 Reproductive Medicine Center, Yuebei People’s Hospital, Shantou University Medical College, Shaoguan, 512026 China; 2 Department of Neonatology, Yuebei People’s Hospital, Shantou University Medical College, Shaoguan, 512026 China; 3 Department of Biobank, Yuebei People’s Hospital, Shantou University Medical College, Shaoguan, 512026 China

**Keywords:** Medical research, Molecular medicine

## Abstract

This study aimed to explore the molecular epidemiology characteristics of deafness susceptibility genes in neonates in northern Guangdong and provide a scientific basis for deafness prevention and control. A total of 10,183 neonates were recruited between January 2018 and December 2022 at Yuebei People's Hospital. Among these, a PCR hybridization screening group of 8276 neonates was tested for four deafness genes: *GJB2, SLC26A4, mtDNA*, and *GJB3* by PCR hybridization. Another group used next-generation sequencing (NGS) to detect genetic susceptibility genes in 1907 neonates. In PCR hybridization screening group, 346 (4.18%) of 8276 neonates were found to be carriers of the deafness gene. Among these, 182 (2.2%) had *GJB2* variants, 114 (1.38%) had *SLC26A4* variants, 35 (0.42%) had *mtDNA* variants, and 15 (0.18%) had *GJB3* variants. In NGS Screening Group, 195 out of 1907 neonates were found to be carriers of the deafness gene, with a positive rate of 10.22%. Among these, 137 (7.18%) had *GJB2* variants, 41 (2.15%) had *SLC26A4* variants, 11 (0.58%) had *mtDNA* variants, and 6 (0.31%) had *GJB3* variants. The prevalence of deafness gene variants was high in Northern Guangdong Province. The most common gene for deafness was *GJB2*, followed by *SLC26A4* and *mtDNA*. *GJB3* variants are rare. Compared with PCR hybridization method, NGS technology can expand the screening scope and greatly improve the detection rate of deafness genes. The c.109G>A of *GJB2* was found to occur at a high frequency, which should be considered. Therefore, it is important to conduct neonatal deafness gene screening to prevent and control hereditary deafness.

## Introduction

According to the World Health Organization (WHO), approximately 500 million people worldwide suffer from disabling deafness. Deafness is the fourth leading cause of disability, accounting for 5.8% of all causes^1^. Ouyang XM reported in 2009 that China's population is approximately 1.3 billion. An estimated 30,000 infants are born with congenital sensorineural hearing loss each year^2^. Approximately 70% of congenital hearing loss cases are estimated to have genetic causes^3^. The deafness genes carried out by our population mainly included *GJB2* (DFNB1A, MIM 220,290)*, SLC26A4* (PDS, MIM 274,600), *mitochondrial DNA* (*mtDNA*), (Mitochondrial disease, MIM 561,000), and *GJB3* (DFNB1A, MIM 220,290)^4^. There are regional, ethnic, and methodological differences between deafness genes^5^. Currently, PCR hybridization method is used to screen for deafness genes in the clinic and only 9–13 variant sites of these four genes are detected^6^. In this study, we used PCR hybridization and next-generation sequencing (NGS) methods to detect deafness susceptibility genes, explore the molecular epidemiological characteristics of deafness genes in neonates in northern Guangdong province, and provide a scientific basis for the accurate prevention and treatment of deafness.

## Materials and methods

### Subjects

A total of 10,183 neonates were enrolled between January 2018 and December 2022 at the Yuebei People's Hospital. Screening of four deafness genes (*GJB2, SLC26A4, mtDNA and GJB3*) without knowing the result of hearing test. Among them, 8276 cases were detected by PCR hybridization, and 1907 cases were detected by NGS. This study was approved by the Ethics Committee of Yuebei People's Hospital (KY-2021-219). Informed consent was obtained from the neonatal guardian, and an informed consent form was signed. All methods followed relevant guidelines and standard operating procedures (SOPs).

### Specimen collection

Dried blood spots from neonates were collected according to the blood collection standards and placed in a sealed bag after natural drying; specimens were sent to the hospital's clinical PCR laboratory department for immediate detection or stored at 2–8 °C.

### PCR hybridization screening

Thirteen variant sites in the four deafness genes were detected using the deafness susceptibility gene kit (Guangdong Kaipu Biotechnology Co., Ltd.). The test kit was approved by the National Medical Products Administration (NMPA, no. 20153401698). *GJB2* included c.35delG, c.155delTCTG, c.176del16, c.235delC, and c.299delAT. *SLC26A4* includes c.1229C>T, c.2168A>G and c.919-2A>G. *mtDNA* includes m.1555A>G, m.1494C>T, m.7445A>G and m.12201T>C. *GJB3* c.538C>T. The laboratory operation and positive judgment standard were based on the kit instructions and SOPs. The PCR amplification system included genomic DNA (2 μL), a PCR reaction solution (27.5 μL), and DNA polymerase (0.5 μL). PCR reaction conditions were as follows: pre-denaturation at 95 °C for 9 min, denaturation at 95 °C for 30 s, annealing at 55 °C for 30 s, elongationat 72 °C for 1 min, 40 cycles, at 72°Cfor 5 min, and storage at 4 °C.

### NGS screening

The target exons of four deafness susceptibility genes were sequenced using the hereditary deafness genetic test kit (BGI Genomics, China). The test kit has been approved by the National Medical Products Administration (NMPA, no. 20203400432). Within ten bases both ends of the exons were analyzed. The basis for estimating the pathogenicity of gene variants is the joint consensus recommendation of the American College of Medical Genetics and Genomics (ACMG) and the Clinical Genome Resource (ClinGen)^7^.

### Statistical analysis

Data analysis was performed using the SPSS22.0 software (IBM, USA). Data are presented as case (n) or percentage (%).

### Ethics statement

This research was approved by the Ethics Committees of Yuebei People’s Hospital.

## Results

### Result of PCR hybridization screening group

**Figure 1:**
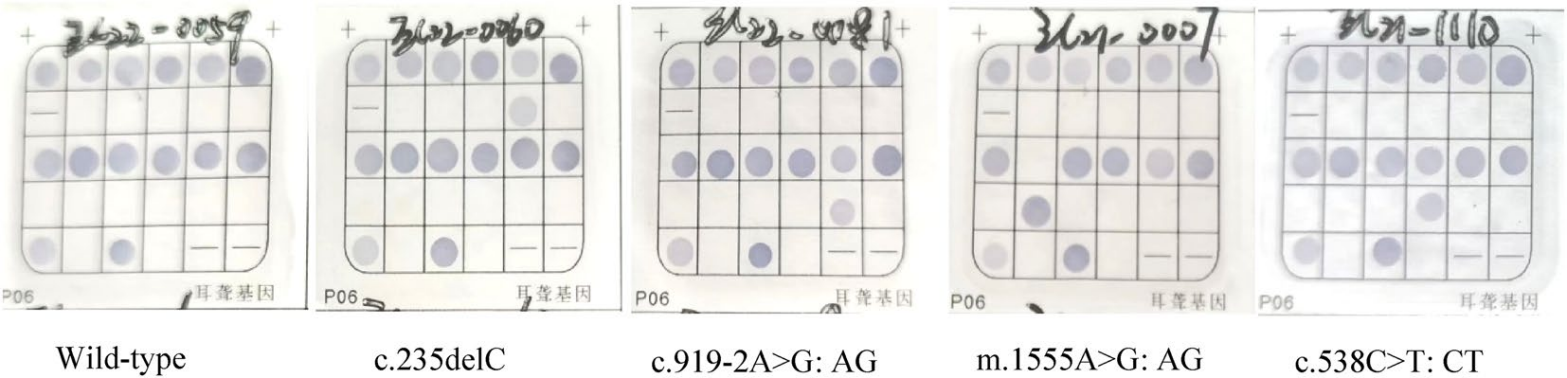
PCR hybridization images.

Among 8276 neonates, 346 cases were found to have deafness gene variants, with a positive rate of 4.18%. Among these, 182 (2.2%, 182/8276) had *GJB2* variants, 114 (1.38%, 114/8276) had *SLC26A4* variants, 35 (0.42%, 35/8276) had *mtDNA* variants, and 15 (0.18%, 15/8276) had *GJB3* variants. The most common deafness gene was *GJB2*, with c.235delC as its hotspot variant, followed by *SLC26A4* and c.919-2A>G, as its hotspot variant (Table 1 and Fig. 1).

**Table 1:** Results of deafness gene in the PCR hybridization screening group (n = 8276).

Gene transcripts ID	Nucleotide change	Amino acid change	Number of cases (het/hom)	Positive rate (%)
*GJB2* NM_004004			**182**	**2.20**
	c.235del C	p.Leu79Cysfs*3	146(146/0)	1.76
	c.299del AT	p.His100Argfs*14	27(27/0)	0.33
	c.176del16	p.Gly59Alafs*18	9(9/0)	0.11
*SLC26A4* NM_000441			**114**	**1.38**
	c.919-2A>G	splice acceptor	87(87/0)	1.05
	c.1229C>T	p.Thr410Met	17(17/0)	0.21
	c.2168A>G	p.His723Arg	10(10/0)	0.12
*mtDNA* NC_012920			**35**	**0.42**
	m.1555A>G	–	25(9/16)	0.30
	m.7445A>G	–	6(0/6)	0.07
	m.1494C>T	–	4(0/4)	0.05
*GJB3* NM_024009	c.538C>T	p.Arg180Ter	**15** 15(15/0)	**0.18** 0.18
Total			**346**	**4.18**

Significant values are in bold.

### Result of NGS screening group

**Figure 2:**
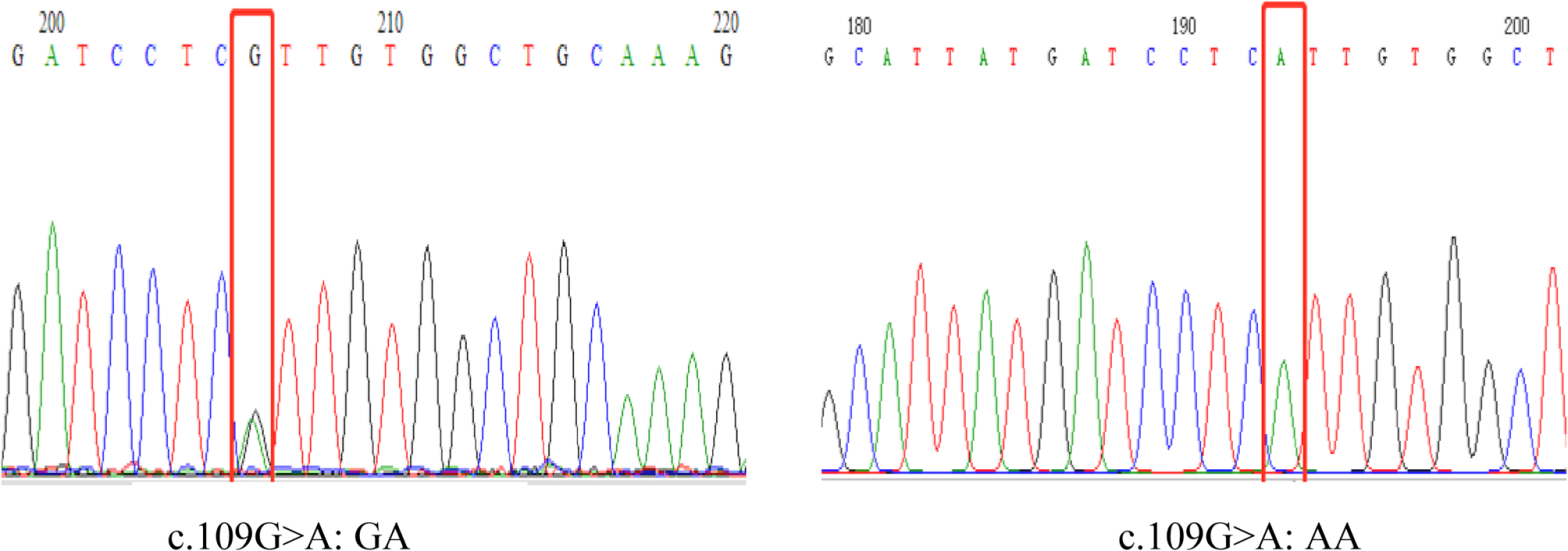
Partial electropherogram of the *GJB2* c.109G>A.

Of the 1907 neonates, 195 were carriers of the deafness gene, with a positivity rate of 10.22%. Among them, 137 (7.18%, 137/1907) had *GJB2* variants, 41 (2.15%, 41/1907) had *SLC26A4* variants, 11 (0.58%, 11/1907) had *mtDNA* variants, and 6 (0.31%, 6/1907) had *GJB3* variants. The most common deafness gene was *GJB2,* with c.109G>A as its hotspot variant, followed by *SLC26A4*, and c.919-2A>G as its hotspot variant (Table 2 and Fig. 2).

**Table 2:** Results of deafness gene in NGS screening group (n = 1907).

Gene transcripts ID	Nucleotide change	Amino acid change	Number of cases (het/hom)	Positive rate (%)
*GJB2*			**137**	**7.18**
NM_004004	c.109G>A	p.Val37Ile	102(97/5)	5.35
	c.235delC	p.Leu79Cysfs*3	30(30/0)	1.57
	c.299_300delAT	p.His100Argfs*14	5(5/0)	0.26
*SLC26A4*			**41**	**2.15**
NM_000441	c.919-2A>G	splice acceptor	30(29/1)	1.57
	c.1229C>T	p.Thr410Met	3(3/0)	0.16
	c.2168A>G	p.His723Arg	2(2/0)	0.10
	c.147C>G	p.Ser49Arg	1(1/0)	0.05
	c.1079C>T	p.Ala360Val	1(1/0)	0.05
	c.1286C>A	p.Ala429Glu	1(1/0)	0.05
	c.1405C>T	p.Pro469Ser	1(1/0)	0.05
	c.1692dupA	p.Cys565Metfs Ter9	1(1/0)	0.05
	c.2086C>T	p.Gln696Ter	1(1/0)	0.05
*mtDNA*			**11**	**0.58**
NC_012920	m.1095T>C	–	7(0/7)	0.37
	m.1555A>G	–	3(0/3)	0.16
	m.1494C>T	–	1(0/1)	0.05
*GJB3*			**6**	**0.31**
NM_024009	538C>T	p.Arg180Ter	4 (4/0)	0.21
	547G>A	p.Glu183Lys	2(2/0)	0.10
Total			**195**	**10.22**

Significant values are in bold.

## Discussion

The detection rate of deafness genes by PCR hybridization screening was only 4.18%, which was lower than the 4.78% of genetic deafness testing results in Chinese newborns^8^. However, that of NGS screening increased to 10.22%, which improved detection efficiency. Both methods found GJB2 to be the most common deafness gene, followed by *SLC26A4* and *mtDNA, GJB3* variants were rare, with positive rates of 2.2%, 1.38%, 0.42%, and 0.18%, respectively, consistent with other reports^9,10^.

*GJB2* is the most common variant associated with hereditary deafness^11^, which encodes the gap junction protein connexin-26 (CX26) on chromosome 13q12.11, which variants and causes congenital sensorineural deafness^12^. The c.235delC was the most common variant in PCR hybridization screening. Significantly, we found that the c.109G>A variant was the most common in NGS screening. It is possible that the c.109G>A variant was not included in PCR hybridization screening. The pathogenicity of the c.109G>A variant is controversial because of the heterogeneity of the hearing phenotype resulting from the variant at this locus. The c.109G>A homozygous variant, which is considered a polymorphism, can also be detected in individuals with normal hearing. Others may even display severe-to-extreme hearing loss^13^. The ClinGen hearing loss expert panel determined that c.109G>A is a causative factor for autosomal recessive non-syndromic hearing loss with variable expression and incomplete penetrance^14^. According to ACMG, the c.109G>A variant is predicted to be a pathogenic variant^15^. In this study, five patients with the c.109G>A homozygous variant and two with heterozygous variants did not pass hearing screening. Further follow-up and investigation are needed to determine whether their hearing can be restored.

*SLC26A4* is the second most mutated gene in hereditary deafness, and c.919-2A>G is a hotspot variant. *SLC26A4* is located on 7q22.3 and encodes the pendrin protein, which causes non-syndromic deafness of the vestibular aqueduct to dilate^16^. Such patients should avoid hard blows, sneezing, blowing nose, head trauma, and other inducements that can effectively prevent deafness^17^.

*MtDNA* variants are one of the causes of drug-induced deafness, leading to cochlear and vestibular cell dysfunction^18^. Using aminoglycosides can lead to deafness. Patients with delayed deafness and their maternal members should be banned from aminoglycoside antibiotics for life^19^. Therefore, screening for deafness genes before aminoglycoside antibiotics can effectively prevent the tragedy of one-needle deafness.

*GJB3* variant is rare, is located on 1p34.3 and encodes gap junction protein 31. Variants cause acquired delayed-onset sensor-neural deafness^20^. The carrier rate of *GJB3* variant is low in China^4,9^. In this study, the variant rate of *GJB3* was also low, at only 0.19%.

Studies have revealed that the results of deafness gene screening can also guide the evaluation of the effectiveness of hearing aids and cochlear implants. For example, patients with deafness associated with *GJB2* or *SLC26A4* have a good prognosis after cochlear implantation^21^. The limitation of this study is insufficient information on the subjects, such as sex, twins, hearing loss, and other clinical features. Making it difficult to directly compare the results with previous data.

## Conclusions

In brief, the key finding of our investigation pertains to the use of NGS, which surpasses the constraints of conventional screening methods in identifying previously overlooked diagnoses. NGS technology has expanded the scope of deafness gene screening and greatly improved the detection rate. Therefore, it is necessary to include deafness gene testing in hearing screening. High-risk groups can be identified early for intervention or diagnostic guidance. This is significant for individualized and accurate prevention and treatment of deafness.

## Data Availability

The original data can be obtained by contacting the corresponding author on reasonable request, or available in the China National Center for Bioinformation, [https://bigd.big.ac.cn/gsa-human]. The assigned accession ID is: HRA006031.
